# Supplementary material for: Epigenetic landscape of the H3K27me3 mark in macrophages transformed by Theileria annulata
Source: Commun Biol. 2026 Feb 24;9:478. doi: 10.1038/s42003-026-09735-3 (PMC13043914; doi:10.1038/s42003-026-09735-3)

Supplementary Information

(6 Figures, Source data for Western blots)

for

Epigenetic landscape of the H3K27me3 mark in macrophages transformed by *Theileria annulata*

Takaya Sakura[1,2,3,4,*], Shahin Tajeri[1,†,*], Zineb Rchiad[1,5], Hifzur R. Ansari[5], Abhinav Kaushik[5], Tobias Mourier[5], Arnab Pain[5,6], Michel Wassef[7,#] and Gordon Langsley[1,2,#]

[1]Biologie Comparative des Apicomplexes, Institut Cochin, Paris, France.
[2]Université de Paris, INSERM U1016, CNRS UMR 8104, France.
[3]Department of Molecular Infection Dynamics, Shionogi Global Infectious Diseases Division, Institute of Tropical Medicine (NEKKEN), Nagasaki University, Sakamoto, Nagasaki 852-8523, Japan.
[4]School of Tropical Medicine and Global Health, Nagasaki University, Nagasaki, 852-8523, Japan.
[5]Pathogen Genomics Laboratory, Biological and Environmental Sciences and Engineering (BESE) Division, King Abdullah University of Science and Technology (KAUST), Saudi Arabia.
[6]International Institute for Zoonosis Control (IIZC), Institute for Vaccine Research and Development (IVReD), Hokkaido University, Sapporo, 001-0020 Japan
[7]Institut Curie, INSERM U934/CNRS UMR 3215, Paris Sciences et Lettres Research University, Sorbonne University, Paris, France

†Current address: Institute for Parasitology and Tropical Veterinary Medicine, Freie Universität Berlin, Berlin, Germany
[*]These authors contributed equally
[#]Corresponding authors

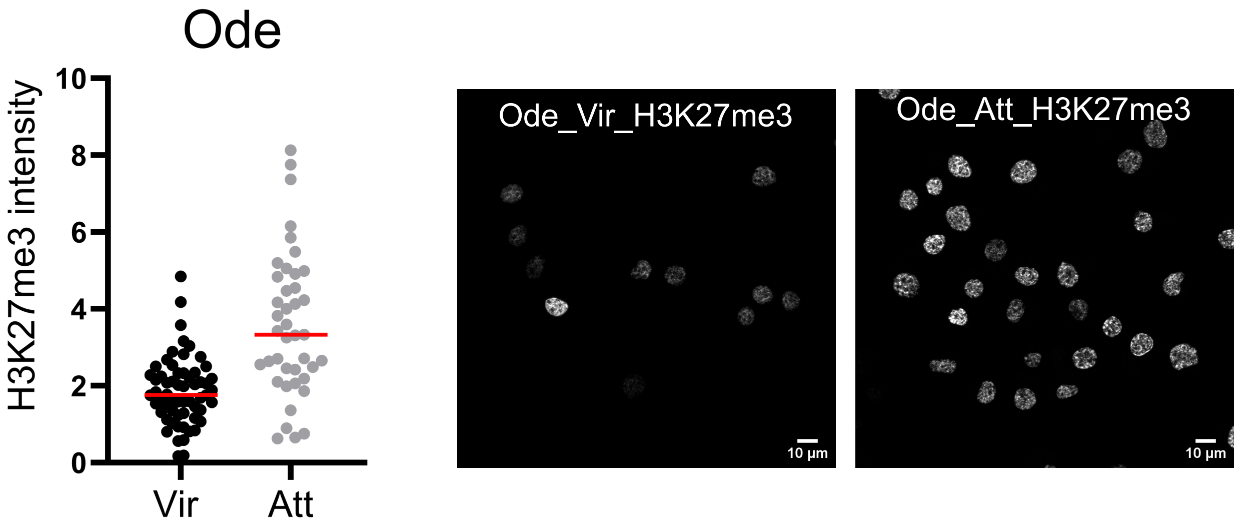

Supplementary Figure 1. Single-cell immunofluorescence quantification of the H3K27me3 mark between Vir and Att Ode macrophages. H3K27me3 levels increased in attenuated macrophages compared with virulent macrophages. Red horizontal bars show the median.

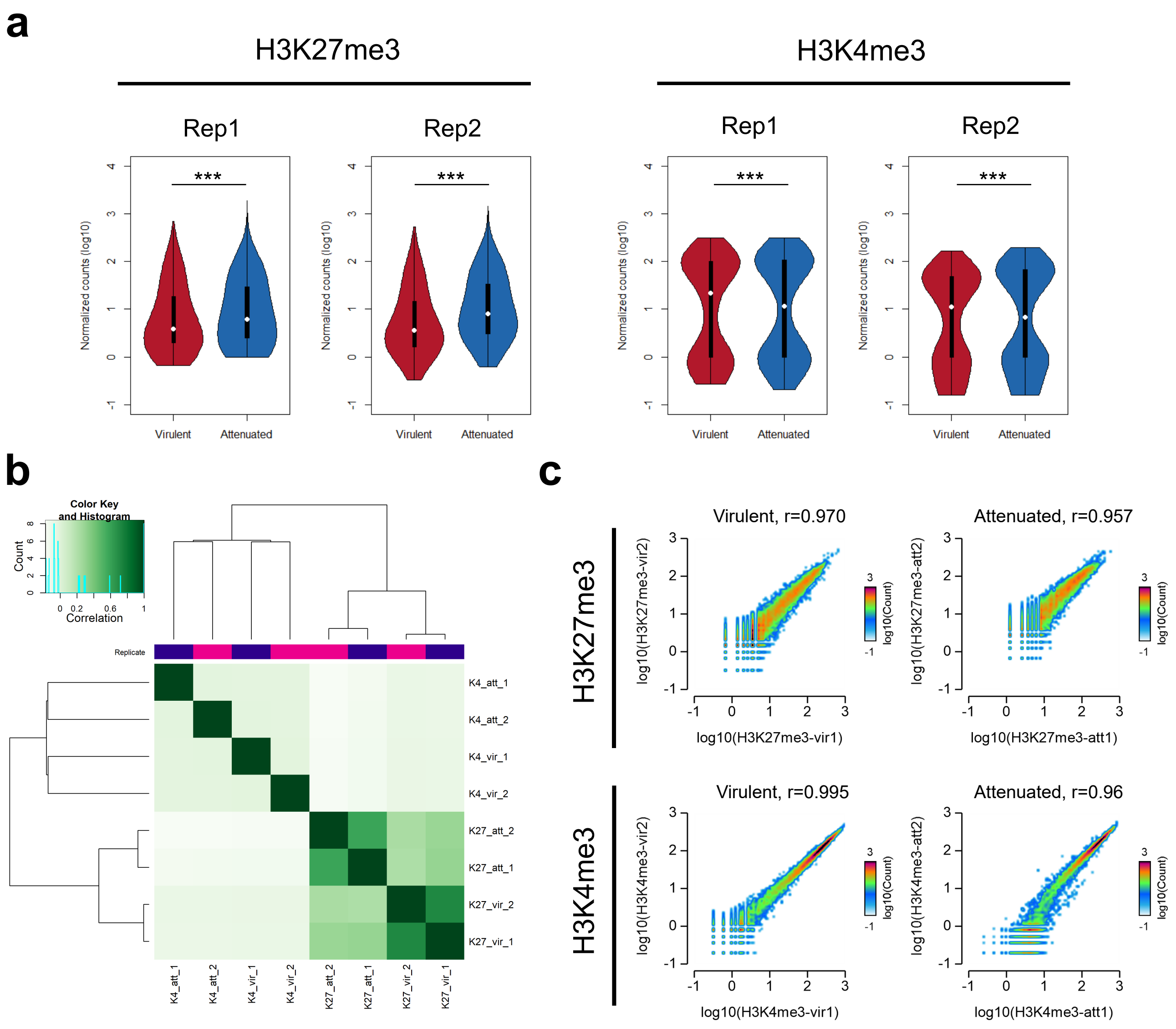

Supplementary Figure 2. Clustering of each ChIP-seq reads and scatter plot. a) Violin plots represent the read counts on TSS around +/- 2kb of each gene obtained from H3K27me3 and H3K4me3 ChIP-seq. ***, Wilcoxon rank-sum test p-value < 2.2e-16. b) Clustering of each ChIP-seq samples visualized by Bioconductor DiffBind package. c) 2D density plots comparing H3K27me3 and H3K4me3 between ChIP-seq biological replicates. Read counts for each gene in replicate 1 (on X-axis) and replicate 2 (on Y-axis) are plotted. The r values represent the Pearson correlation coefficients.

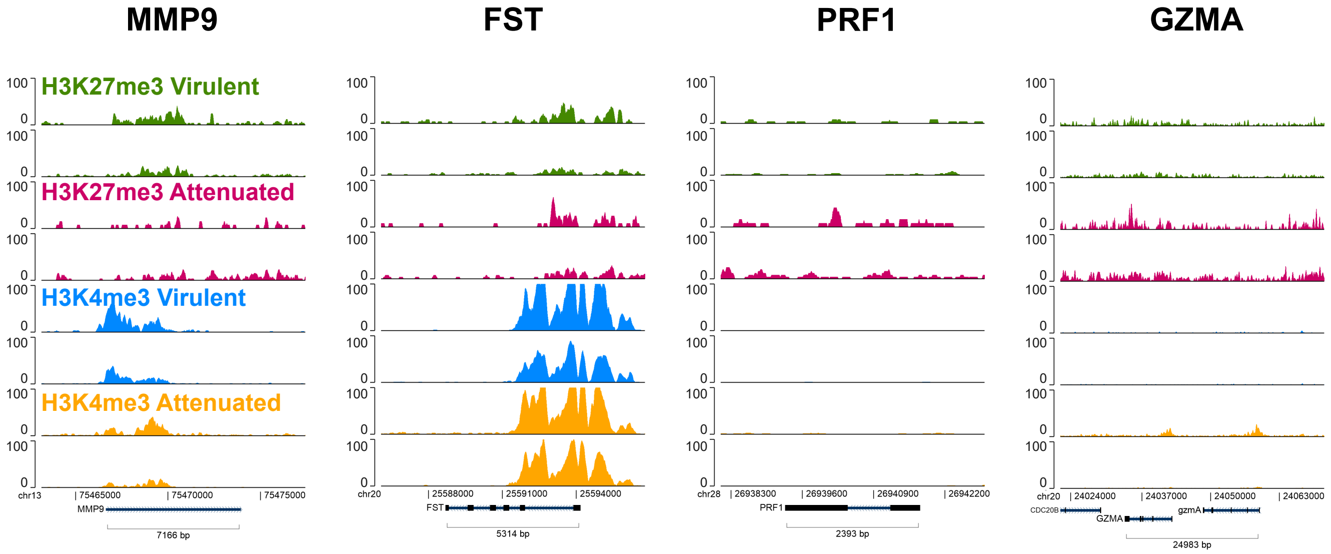

Supplementary Figure 3. Genomic distribution of H3K27me3 and H3K4me3 marks around 4 representative genes of the 21 common upregulated genes relative to Figure 5. Profile of each mark on the MMP9, FST, PRF1, GZMA genes.

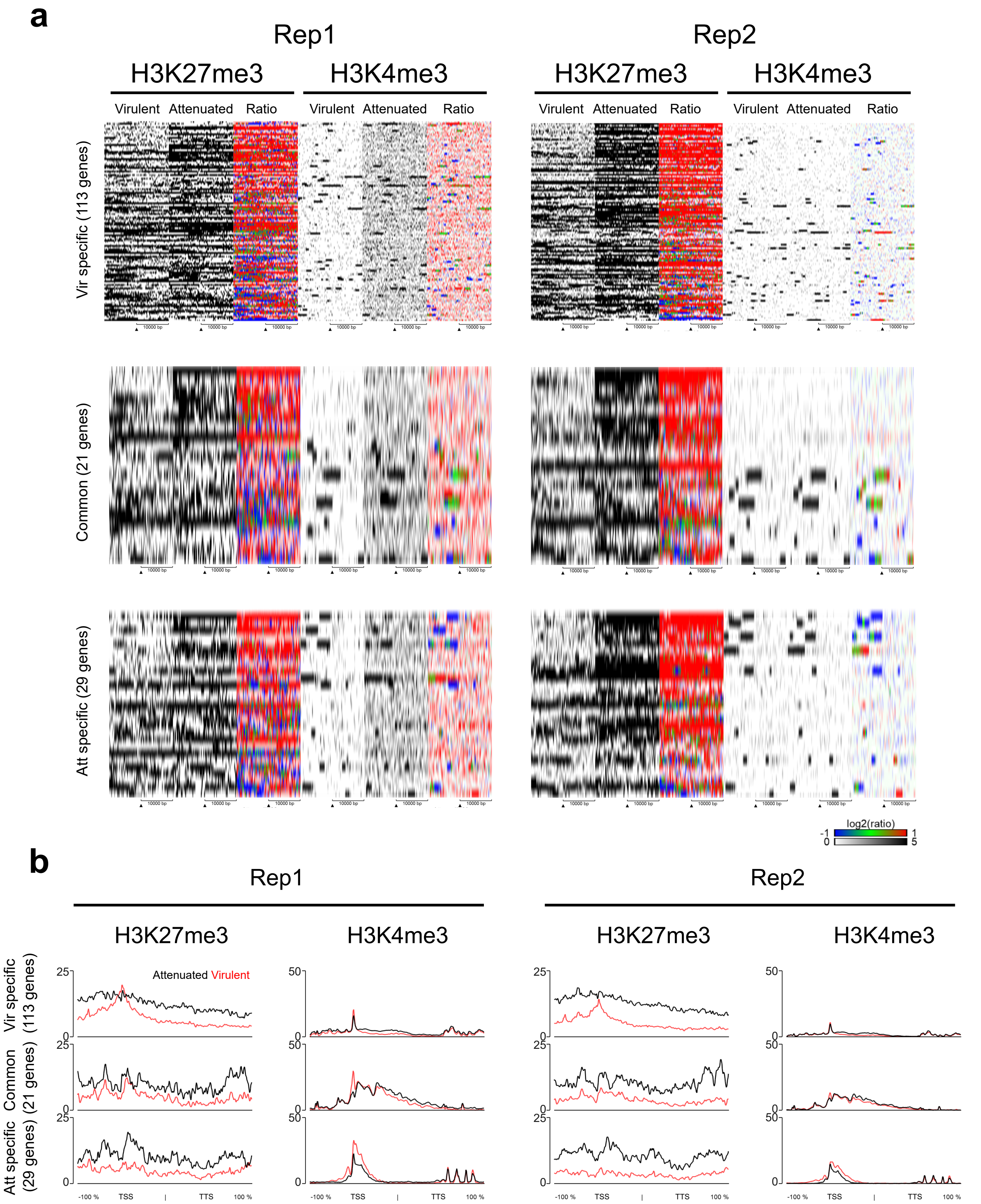

Supplementary Figure 4. H3K27me3 and H3K4me3 profiles of upregulated genes upon PRC2 inhibitor related to Fig. 5b. a) H3K27me3 and H3K4me3 heat maps around TSS +/- 10 kb of 3 groups of genes related to Fig.5b in virulent or attenuated macrophages. Genes were sorted by the ratio of H3K27me3 signal log2 (Att/Vir). b) Average H3K27me3 and H3K4me3 signal intensities along the 3 groups of genes defined in Fig.5b in virulent or attenuated macrophages. The X-axis represents a window centered on each gene, spanning from the TSS to the TTS with an additional ±100% flanking region. Black lines represent signals from virulent macrophages, whereas red lines represent those from attenuated macrophages.

a

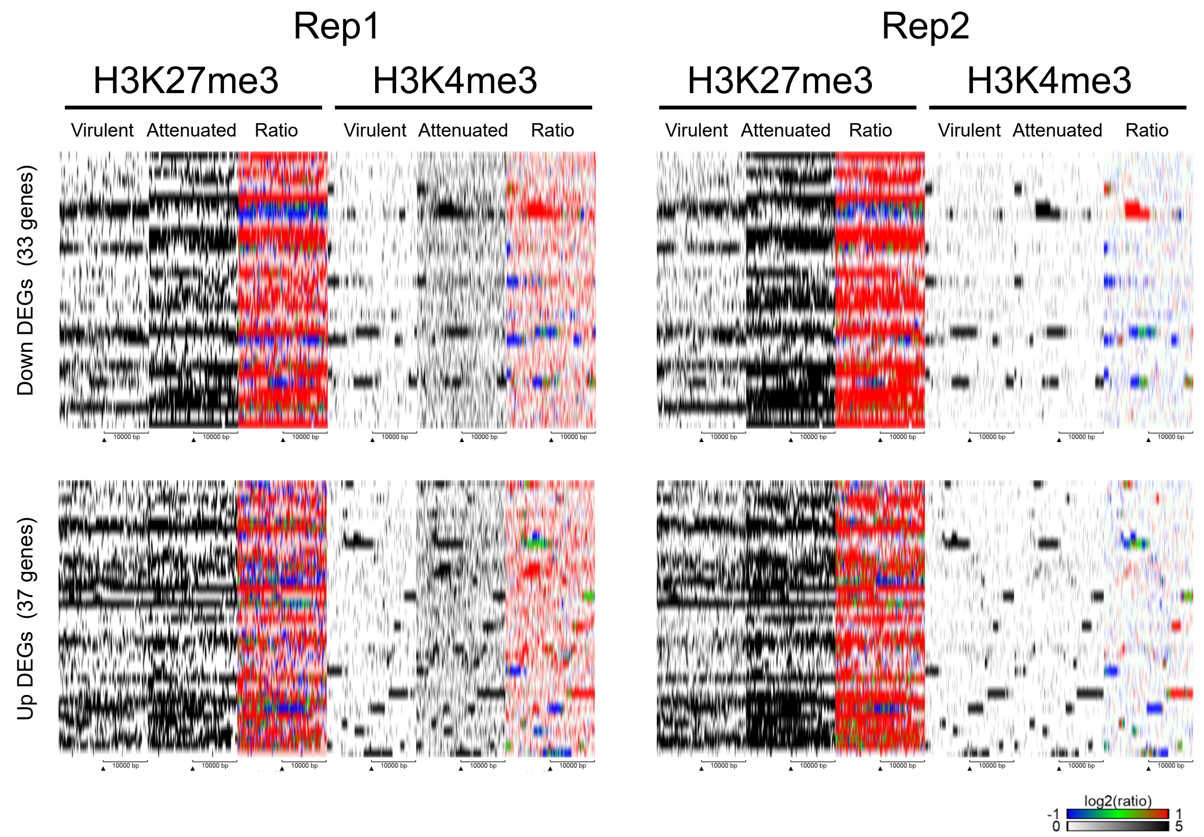

b

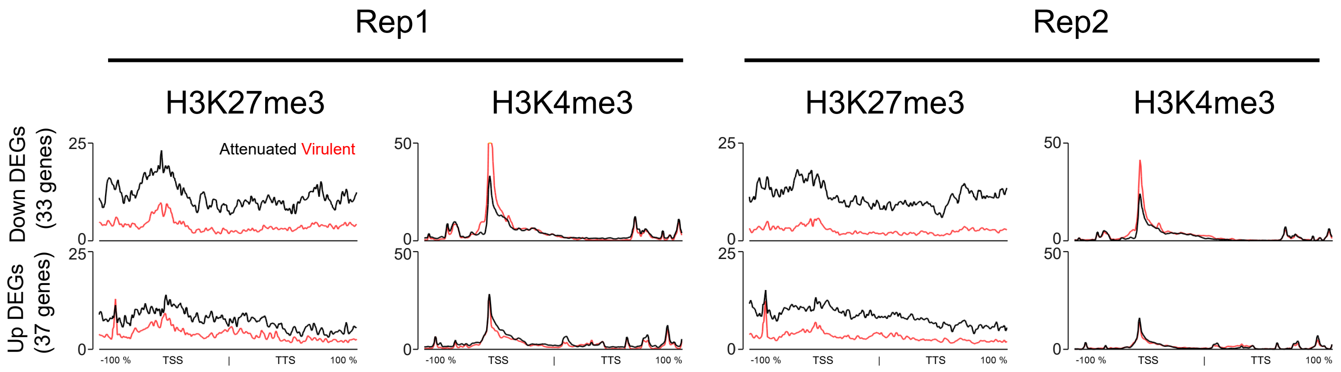

Supplementary Figure 5. H3K27me3 and H3K4me3 profiles of Down and Up DEGs in attenuated macrophages. a) H3K27me3 and H3K4me3 heat maps around TSS +/- 10 kb of Down and Up DEGs in attenuated macrophages. b) Average H3K27me3 and H3K4me3 signal intensities along genes that are significantly up- or downregulated in attenuated macrophages. The X-axis represents a window centered on each gene, spanning from the TSS to the TTS with an additional ±100% flanking region. Black lines represent signals from virulent macrophages, whereas red lines represent those from attenuated macrophages.

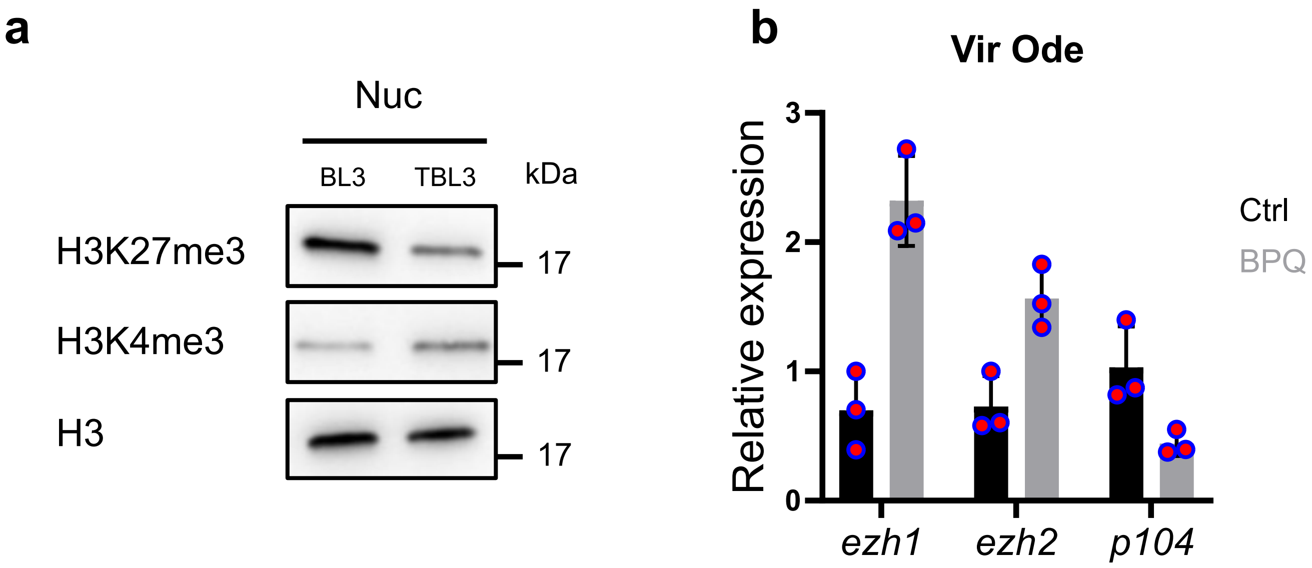

Supplementary Figure 6. *Theileria annulata* infection downregulates PRC2 complex function. a) Western blot analysis of nuclear extracts from uninfected (BL3) and infected (TBL3) bovine B cells shows a global decrease in H3K27me3 levels in TBL3. b) Elimination of the parasite by treating *T. annulata*-transformed macrophages (TaC12) with buparvaquone (BPQ) results in increased *ezh1* and *ezh2* mRNA levels, supporting the data shown in panel (a). Expression of the *p104* gene encoding a major *T. annulata* schizont surface protein was also measured to demonstrate the effect of BPQ on parasite clearance.

Source data for Figure 2a

H3K27me1

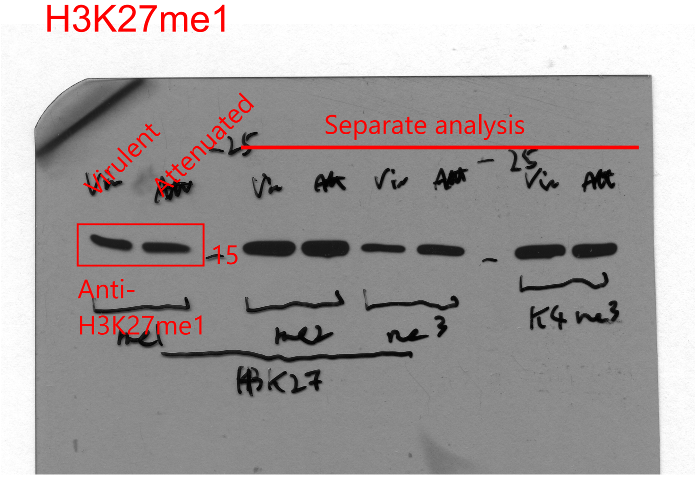

H3K27me3

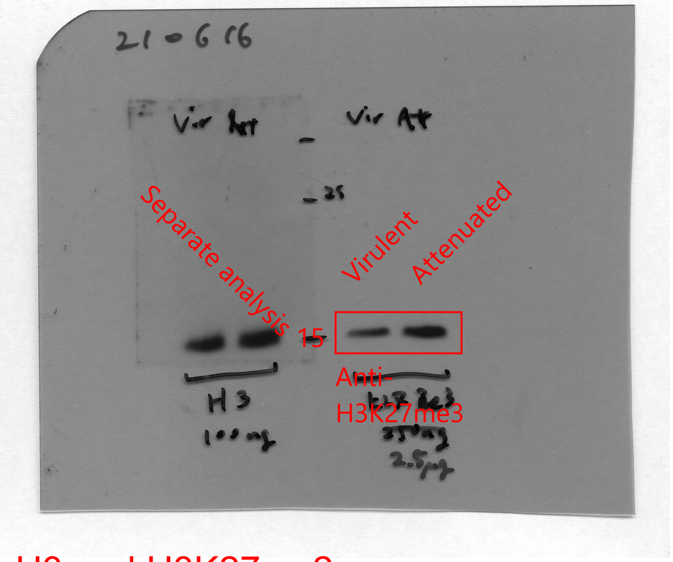

H3K4me3

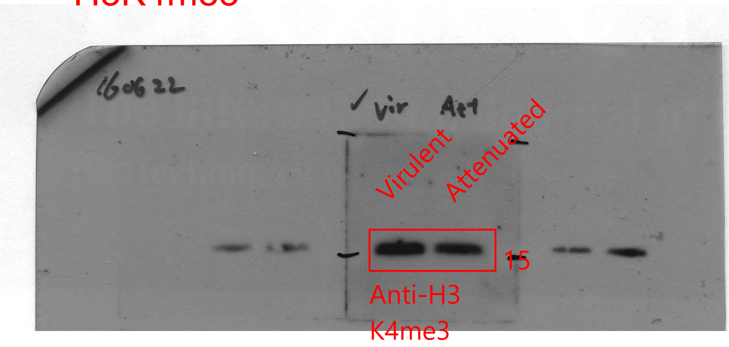

H3 and H3K27me2

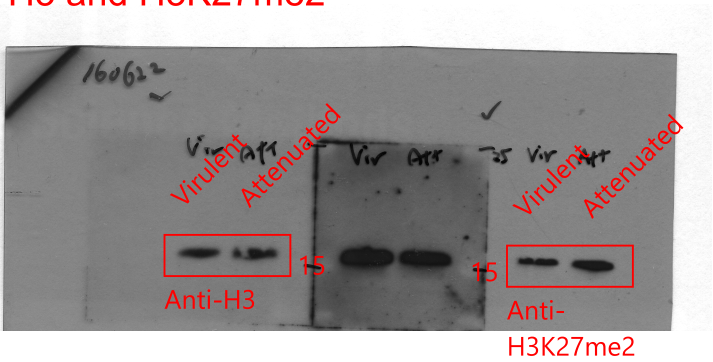

Ezh2

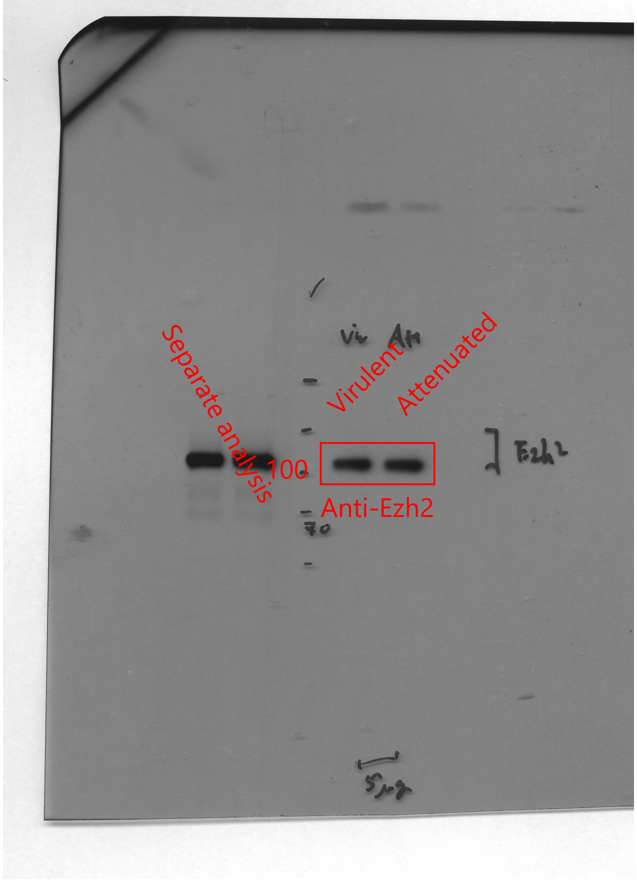

Source data for Figure 4a

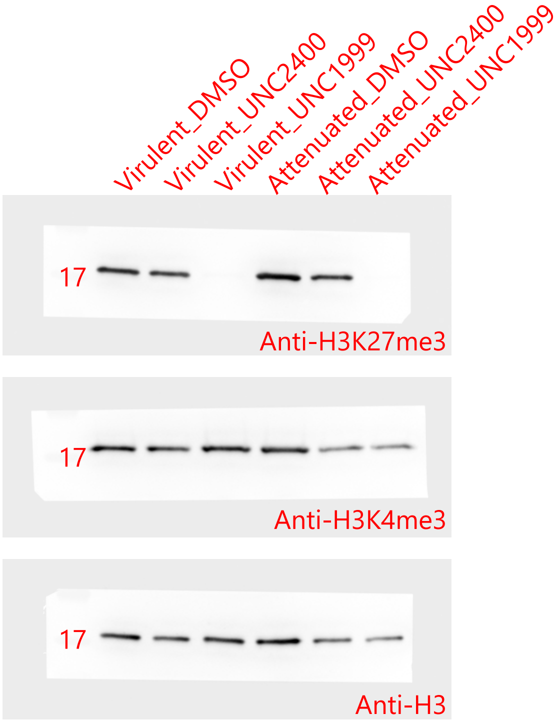

Source data for Supplementary Figure 6a

Exposure 10 sec: H3K27me3

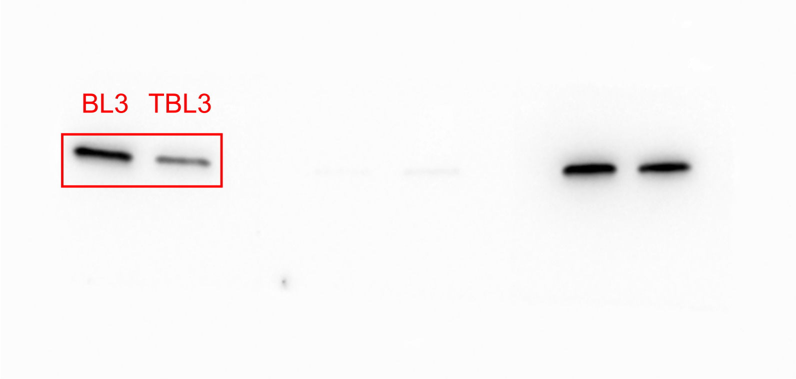

Exposure 9 sec: H3

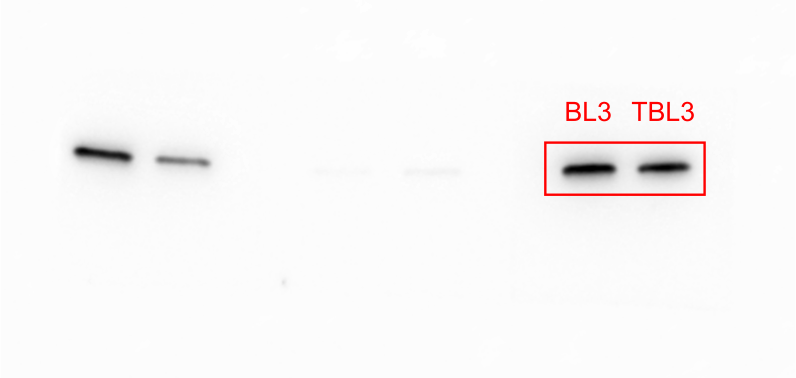

Exposure 3 min: H3K4me3

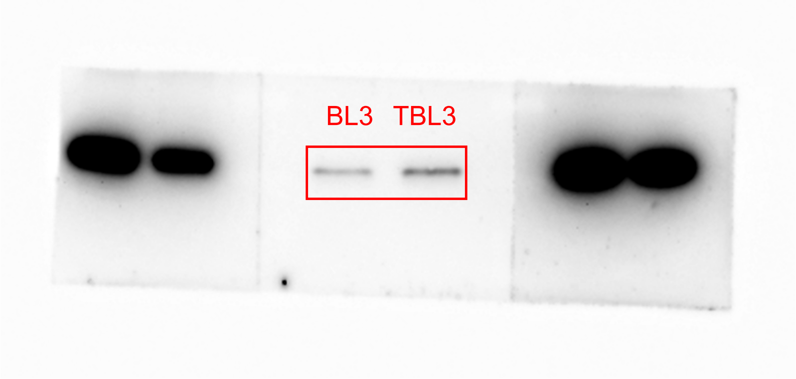

Exposure 400 msec: Marker

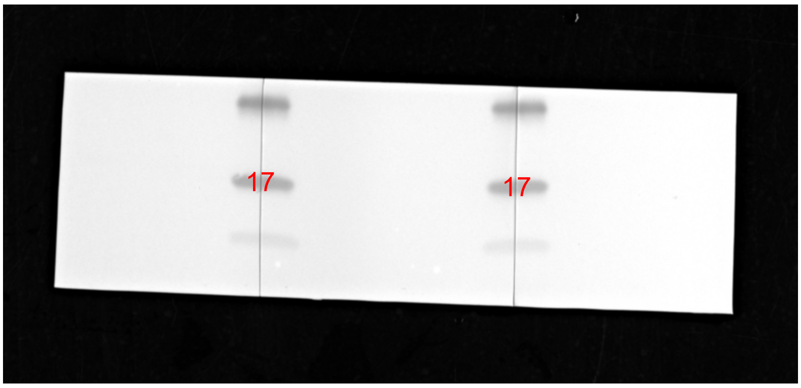

Supplement: Supplementary file 2 — Supplementary Information [file 42003_2026_9735_MOESM2_ESM.pdf]
