# Supplementary material for: Epigenetic landscape of the H3K27me3 mark in macrophages transformed by Theileria annulata
Source: Commun Biol. 2026 Feb 24;9:478. doi: 10.1038/s42003-026-09735-3 (PMC13043914; doi:10.1038/s42003-026-09735-3)
Supplement: Supplementary file 3 — Description of Additional Supplementary files [file 42003_2026_9735_MOESM3_ESM.pdf]

Description of Additional Supplementary files

Supplementary Data 1. List of differentially expressed genes corresponding to Fig 5b

Supplementary Data 2. List of GO terms of DEGs corresponding to Fig 5c

Supplementary Data 3. Summary of ChIP-seq experiments

Supplementary Data 4. List of primers for qRT-PCR

Supplementary Data 5. Source data are provided in Supplementary Data 5**.**
