# Supplementary material for: Epigenetic landscape of the H3K27me3 mark in macrophages transformed by Theileria annulata
Source: Commun Biol. 2026 Feb 24;9:478. doi: 10.1038/s42003-026-09735-3 (PMC13043914; doi:10.1038/s42003-026-09735-3)
Supplement: Supplementary file 9 — Reporting Summary [file 42003_2026_9735_MOESM9_ESM.pdf]

nature portfolio

Corresponding author(s): Gordon Langsley & Michel Wassef

Last updated by author(s): 24/12/2025

Reporting Summary

Nature Portfolio wishes to improve the reproducibility of the work that we publish. This form provides structure for consistency and transparency in reporting. For further information on Nature Portfolio policies, see our Editorial Policies and the Editorial Policy Checklist.

Statistics

For all statistical analyses, confirm that the following items are present in the figure legend, table legend, main text, or Methods section.

n/a	Confirmed	
☐	☒	The exact sample size (*n*) for each experimental group/condition, given as a discrete number and unit of measurement
☐	☒	A statement on whether measurements were taken from distinct samples or whether the same sample was measured repeatedly
☐	☒	The statistical test(s) used AND whether they are one- or two-sided<br>*Only common tests should be described solely by name; describe more complex techniques in the Methods section.*
☒	☐	A description of all covariates tested
☒	☐	A description of any assumptions or corrections, such as tests of normality and adjustment for multiple comparisons
☐	☒	A full description of the statistical parameters including central tendency (e.g. means) or other basic estimates (e.g. regression coefficient) AND variation (e.g. standard deviation) or associated estimates of uncertainty (e.g. confidence intervals)
☐	☒	For null hypothesis testing, the test statistic (e.g. *F*, *t*, *r*) with confidence intervals, effect sizes, degrees of freedom and *P* value noted<br>*Give P values as exact values whenever suitable.*
☒	☐	For Bayesian analysis, information on the choice of priors and Markov chain Monte Carlo settings
☒	☐	For hierarchical and complex designs, identification of the appropriate level for tests and full reporting of outcomes
☒	☐	Estimates of effect sizes (e.g. Cohen's *d*, Pearson's *r*), indicating how they were calculated

Our web collection on statistics for biologists contains articles on many of the points above.

Software and code

Policy information about availability of computer code

Data collection	Leica DMI6000 (Leica); Illumina Hiseq2500 (Illumina, Inc.) for ChIP-seq; Illumina Hiseq4000 for RNA-seq ; Fusion FX (Vilber Lourmat); LightCycler480 Instrument (Roche Diagnostics)
Data analysis	Trimmomatic (v0.36)<br>Bowtie2 (v2.1.0)<br>Hisat2 (v2.1.0)<br>Samtools (v1.10)<br>HTseq count (v0.9.1)<br>NetworkAnalyst (v2.0)<br>Easeq (v1.0, v1.2)<br>R (v4.0.2, v4.1.0)<br>IGV (v2.4.19)<br>MACS2 (v2.1.1.20160309)<br>DiffBind (v2.4.1)<br>AgriGO (v2.0)<br>clusterProfiler (v4.16.0)

For manuscripts utilizing custom algorithms or software that are central to the research but not yet described in published literature, software must be made available to editors and reviewers. We strongly encourage code deposition in a community repository (e.g. GitHub). See the Nature Portfolio guidelines for submitting code & software for further information.

Data

Policy information about availability of data

All manuscripts must include a data availability statement. This statement should provide the following information, where applicable:

- Accession codes, unique identifiers, or web links for publicly available datasets
- A description of any restrictions on data availability
- For clinical datasets or third party data, please ensure that the statement adheres to our policy

RNAseq and ChIP-seq data for this study have been deposited in the European Nucleotide Archive (ENA) under accession no. PRJEB52136 which will become pubic upon acceptance of the manuscript.
The following reference genomes and data set were used for data analysis in this study;
Bos Taurus: Cow-bosTau8
Drosophila melanogaster: Droso-dmel5.41
RNAseq data of the comparison between virulent and attenuated Ode macrophages: PMID: 32830401; NCBI Gene Expression Omnibus, GEO ID: GSE135377.
https://www.ncbi.nlm.nih.gov/geo/query/acc.cgi?acc=GSE135377

Research involving human participants, their data, or biological material

Policy information about studies with human participants or human data. See also policy information about sex, gender (identity/presentation), and sexual orientation and race, ethnicity and racism.

Reporting on sex and gender	N/A
Reporting on race, ethnicity, or other socially relevant groupings	N/A
Population characteristics	N/A
Recruitment	N/A
Ethics oversight	N/A

Note that full information on the approval of the study protocol must also be provided in the manuscript.

Field-specific reporting

Please select the one below that is the best fit for your research. If you are not sure, read the appropriate sections before making your selection.

☒ Life sciences ☐ Behavioural & social sciences ☐ Ecological, evolutionary & environmental sciences

For a reference copy of the document with all sections, see nature.com/documents/nr-reporting-summary-flat.pdf

Life sciences study design

All studies must disclose on these points even when the disclosure is negative.

Sample size	Sample size for mice experiments (four groups of five) was based on previously published studies by our group (PMID: 16778183, PMID: 25375322) and are similar sample sizes used in other studies.
Data exclusions	No data were excluded.
Replication	To ensure reproducibility qRT-PCR experiments, experiments were replicated in two independent laboratories with different PCR machines and PCR reagents.
Randomization	For mice experiments animals were assigned to groups manually (5 mice per group) considering even distribution of sex and age across all groups.
Blinding	Blinding was not used for mouse injections, or daily oral administration of the PRC2 inhibitor treatment.

Reporting for specific materials, systems and methods

We require information from authors about some types of materials, experimental systems and methods used in many studies. Here, indicate whether each material, system or method listed is relevant to your study. If you are not sure if a list item applies to your research, read the appropriate section before selecting a response.

Materials & experimental systems

n/a	Involved in the study
☐	☒ Antibodies
☐	☒ Eukaryotic cell lines
☒	☐ Palaeontology and archaeology
☐	☒ Animals and other organisms
☒	☐ Clinical data
☒	☐ Dual use research of concern
☒	☐ Plants

Methods

n/a	Involved in the study
☐	☒ ChIP-seq
☒	☐ Flow cytometry
☒	☐ MRI-based neuroimaging

Antibodies

Antibodies used

All antibodies used were either commercial, and when not, had been previously published and referenced. For example, the anti-T. annulata monoclonal antibody against p104 was the 1C12 monoclonal antibody described in (B. R. Shiels, McDougall, Tait, & Brown, 1986).
H3K27me1 mouse monoclonal Active Motif 0321
H3K27me2 mouse monoclonal Active Motif 0324
H3K27me3 rabbit monoclonal CST C36B11
H3K4me3 rabbit monoclonal CST C42D8
H3 rabbit polyclonal Active Motif #39163
EZH2 antibody rabbit polyclonal homemade

Validation

The parasite specificity of the monoclonal antibody 1C12 was validated in (B. R. Shiels, McDougall, Tait, & Brown, 1986).
H3K27me1: https://www.activemotif.com/catalog/details/61015
H3K27me2: https://www.activemotif.com/catalog/details/61435/histone-h3k27me2-antibody-mab-clone-mabi0324
H3K27me3: https://www.cellsignal.com/products/primary-antibodies/tri-methyl-histone-h3-lys27-c36b11-rabbit-monoclonal-antibody/9733
H3K4me3: https://www.cellsignal.com/products/primary-antibodies/tri-methyl-histone-h3-lys4-c42d8-rabbit-monoclonal-antibody/9751
H3: https://www.citeab.com/antibodies/82319-39163-histone-h3-c-terminal-antibody-pab
EZH2 antibody rabbit polyclonal homemade (Margueron R, et al. (2008) Ezh1 and Ezh2 Maintain Repressive Chromatin through Different Mechanisms.)

Eukaryotic cell lines

Policy information about cell lines and Sex and Gender in Research

Cell line source(s)

The virulent and attenuated Ode cells lines were first isolate by D.K. Singh, N.D.D.B., Anand, India and given to R. Hall and G. Langsley and described in Loss of matrix metalloproteinase 9 activity in Theileria annulata-attenuated cells is at the transcriptional level and is associated with differentially expressed AP-1 species.
Adamson R, Logan M, Kinnaird J, Langsley G, Hall R.
Mol Biochem Parasitol. 2000 Feb 25;106(1):51-61. doi: 10.1016/s0166-6851(99)00213-3.
PMID: 10743610.
The TBL3 and BL3 lines used in this study have been previously characerised and validated by us (PMID: 9747972, PMID: 16761111).

Authentication

Loss of matrix metalloproteinase 9 activity in Theileria annulata-attenuated cells is at the transcriptional level and is associated with differentially expressed AP-1 species.
Adamson R, Logan M, Kinnaird J, Langsley G, Hall R.
Mol Biochem Parasitol. 2000 Feb 25;106(1):51-61. doi: 10.1016/s0166-6851(99)00213-3.
PMID: 10743610.

Mycoplasma contamination

All cells lines were sroutinely tested for Mycoplasma contamination and found to be negative.

Commonly misidentified lines
(See ICLAC register)

Name any commonly misidentified cell lines used in the study and provide a rationale for their use.

Animals and other research organisms

Policy information about studies involving animals; ARRIVE guidelines recommended for reporting animal research, and Sex and Gender in Research

Laboratory animals

Twenty Rag2gammaC mice (Mus musculus) were divided into four groups of five to study the dissemination potential of Theileria annulata-transformed macrophages. All groups were age- and sex-matched. The age range of the study animals was 43–66 days.

Wild animals

N/A

Reporting on sex

Our findings were not specific to one sex, but the study groups were age-matched. Sex assignment was based on external genitalia

Reporting on sex	examination at birth or shortly after. The presence of scrotal sacs as well as anogenital distance was considered. We have not studied the effect of sex and age on the dissemination of Theileria annulata-transformed macrophages; therefore, we matched the groups by age and sex.
Field-collected samples	N/A
Ethics oversight	All mouse experiments were approved (number CEEA34.GL.03312) by the ethics committee for animal experimentation at the Université Paris-Descartes, now called Université Paris-Cité. The university ethics committee is registered with the French National Ethics Committee for Animal Experimentation that itself is registered with the European Ethics Committee for Animal Experimentation. The right to perform the mice experiments was obtained from the French National Service for the Protection of Animal Health and satisfied the animal welfare conditions defined by laws (R214-87 to R214-122 and R215-10) and GL was responsible for all experiment as he holds the French National Animal Experimentation permit with the authorisation number (B-75-1249).

Note that full information on the approval of the study protocol must also be provided in the manuscript.

Plants

Seed stocks	N/A
Novel plant genotypes	N/A
Authentication	N/A

ChIP-seq

Data deposition

☒ Confirm that both raw and final processed data have been deposited in a public database such as GEO.

☒ Confirm that you have deposited or provided access to graph files (e.g. BED files) for the called peaks.

Data access links
May remain private before publication.

RNAseq and ChIP-seq data for this study have been deposited in the European Nucleotide Archive (ENA) under accession no. PRJEB52136.

Files in database submission

RNAseq raw files:
M_17_5851_DA1_AD005_L007_R2_001.fastq.gz
M_17_5852_DA2_AD006_L007_R1_001.fastq.gz
M_17_5852_DA2_AD006_L007_R2_001.fastq.gz
M_17_5853_DA6_AD007_L007_R1_001.fastq.gz
M_17_5853_DA6_AD007_L007_R2_001.fastq.gz
M_17_5848_DV2_AD002_L007_R1_001.fastq.gz
M_17_5848_DV2_AD002_L007_R2_001.fastq.gz
M_17_5849_DV4_AD003_L007_R1_001.fastq.gz
M_17_5849_DV4_AD003_L007_R2_001.fastq.gz
M_17_5850_DV6_AD004_L007_R1_001.fastq.gz
M_17_5850_DV6_AD004_L007_R2_001.fastq.gz
M_17_5851_DA1_AD005_L007_R1_001.fastq.gz
M_17_5858_19A2_AD012_L008_R2_001.fastq.gz
M_17_5859_19A6_AD013_L008_R1_001.fastq.gz
M_17_5859_19A6_AD013_L008_R2_001.fastq.gz
M_17_5854_19V2_AD008_L008_R1_001.fastq.gz
M_17_5854_19V2_AD008_L008_R2_001.fastq.gz
M_17_5855_19V4_AD009_L008_R1_001.fastq.gz
M_17_5855_19V4_AD009_L008_R2_001.fastq.gz
M_17_5856_19V6_AD010_L008_R1_001.fastq.gz
M_17_5856_19V6_AD010_L008_R2_001.fastq.gz
M_17_5857_19A1_AD011_L008_R1_001.fastq.gz
M_17_5857_19A1_AD011_L008_R2_001.fastq.gz
M_17_5858_19A2_AD012_L008_R1_001.fastq.gz
M_17_5862_24V6_AD016_L007_R1_001.fastq.gz
M_17_5862_24V6_AD016_L007_R2_001.fastq.gz
M_17_5863_24A1_AD018_L007_R1_001.fastq.gz
M_17_5863_24A1_AD018_L007_R2_001.fastq.gz
M_17_5864_24A2_AD019_L007_R1_001.fastq.gz
M_17_5864_24A2_AD019_L007_R2_001.fastq.gz
M_17_5865_24A6_AD020_L007_R1_001.fastq.gz

M_17_5865_24A6_AD020_L007_R2_001.fastq.gz
M_17_5860_24V2_AD014_L007_R1_001.fastq.gz
M_17_5860_24V2_AD014_L007_R2_001.fastq.gz
M_17_5861_24V4_AD015_L007_R1_001.fastq.gz
M_17_5861_24V4_AD015_L007_R2_001.fastq.gz
ChIP-seq raw files:
A593C1.R1.fastq.gz
A593C2.R1.fastq.gz
A593C3.R1.fastq.gz
A593C4.R1.fastq.gz
A593C5.R1.fastq.gz
A593C6.R1.fastq.gz
A593C7.R1.fastq.gz
A593C8.R1.fastq.gz
ChIP-seq Peaks files:
Peaks from K4-att1 using Input-att as negative control.bed
Peaks from K4-att2 using Input-att as negative control.bed
Peaks from K4-vir1 using Input-vir as negative control.bed
Peaks from K4-vir2 using Input-vir as negative control.bed
Peaks from K27-att1 using Input-att as negative control.bed
Peaks from K27-att2 using Input-att as negative control.bed
Peaks from K27-vir1 using Input-vir as negative control.bed
Peaks from K27-vir2 using Input-vir as negative control.bed

Genome browser session (e.g. UCSC)

No longer applicable

Methodology

Replicates

Two independent biological replicates of virulent and attenuated macrophages were performed for each ChIP-seq experiment using H3K4me3 and H3K27me3 antibodies. The replicates were consistent, as shown in the Supplementary Fig. 2.

Sequencing depth

A593C1 K4-vir-no1 Total reads: 32,469,115 Mapped reads: 12,583,269 Length of reads: 100 single-end
A593C2 K4-vir-no2 Total reads: 36,167,184 Mapped reads: 11,276,896 Length of reads: 100 single-end
A593C3 K4-att-no1 Total reads: 44,483,848 Mapped reads: 28,076,449 Length of reads: 100 single-end
A593C4 K4-att-no2 Total reads: 32,716,931 Mapped reads: 12,923,742 Length of reads: 100 single-end
A593C5 K27-vir-no1 Total reads: 18,050,120 Mapped reads: 15,978,932 Length of reads: 100 single-end
A593C6 K27-vir-no2 Total reads: 28,182,272 Mapped reads: 24,459,519 Length of reads: 100 single-end
A593C7 K27-att-no1 Total reads: 18,941,298 Mapped reads: 17,603,928 Length of reads: 100 single-end
A593C8 K27-att-no2 Total reads: 39,697,626 Mapped reads: 37,243,588 Length of reads: 100 single-end
A593C9 Input-vir-no2 Total reads: 52,213,479 Mapped reads: 49,541,870 Length of reads: 100 single-end
A593C10 Input-att-no2 Total reads: 73,859,203 Mapped reads: 70,554,925 Length of reads: 100 single-end

Antibodies

Antibody Host Source Clone/identifier
H3K27me3 rabbit monoclonal CST C36B11
H3K4me3 rabbit monoclonal CST C42D8

Peak calling parameters

Total precipitated reads were mapped to the Bos taurus (bosTau8) and Drosophila melanogaster (Droso-dmel5.41) by bowtie2 (v2.1.0) with the method: Global alignment - 1 mismatch in seed alignment (of size 22). The normalization factor of each sample was calculated by the formula: "Normalization factor=1/(Number of mapped reads to Droso genome)", according to the spiking methods (Supplementary Table 1 and ref. 34).
Peak calling was performed using Adaptive Local Threshold (ALT) equipped in Easeq (v1.0) (window size: 3000bp for H3K27me3 and 1500bp for H3K4me3, p-value:1E-5, false discovery rate (FDR):1E-5, Log2fold diff :2, merge within:100bp).

Data quality

Methods used to ensure data quality are detailed just above in "Peak calling parameters." All peaks fulfill the FDR 5% cutoff. Numbers of peaks called using EaSeq above 5-fold enrichment (Log2 fold diff = 2.322) are as follows:
H3K27-vir1: 32297 of 32493 unmerged peaks
H3K27-vir2: 35895 of 36164 unmerged peaks
H3K27-att1: 43560 of 43792 unmerged peaks
H3K27-att1: 49735 of 50381 unmerged peaks
H3K4-vir1: 21148 of 22270 unmerged peaks
H3K4-vir2: 22331 of 23476 unmerged peaks
H3K4-att1: 25142 of 25144 unmerged peaks
H3K4-att2: 18532 of 19661 unmerged peaks

Software

All software used to analyze the ChIP-seq data is described in “Data analysis” and "Peak calling parameters."
